# Evaluation of Electronic Service-Learning (e-Service-Learning) Projects in Mainland China under COVID-19

**DOI:** 10.1007/s11482-022-10058-8

**Published:** 2022-05-13

**Authors:** Daniel T. L. Shek, Xiang Li, Lu Yu, Li Lin, Yikang Chen

**Affiliations:** 1 Department of Applied Social Sciences, The Hong Kong Polytechnic University, Hung Hom, Hong Kong; 2 School of Graduate Studies and Department of Applied Psychology, Lingnan University, Tuen Mun, Hong Kong

**Keywords:** Service-learning, Program evaluation, Effectiveness, University students, Chinese context

## Abstract

The use of electronic service-learning (e-Service-Learning or e-SL) is valuable under COVID-19 because we can provide the service without physical contact. Unfortunately, evaluation of e-SL is not widespread and there is no known study in different Chinese societies. Besides, there are many methodological limitations of the existing studies in the field. In this paper, we evaluated e-SL projects implemented in summer 2020 and 2021 in three sites in China. First, we examined service providers’ changes based on pretest and posttest scores (i.e., objective outcome evaluation) and their perceptions of the e-SL projects (i.e., subjective outcome evaluation based on the service providers). Second, graduate student assessors in Chinese mainland universities and teachers of primary school students (i.e., service recipients) rated the SL program quality, service providers’ performance and benefits to the service recipients after program completion (i.e., subjective outcome evaluation of SL projects based on other stakeholders). Third, trained graduate student assessors evaluated service quality during the implementation process (i.e., process evaluation). We found that university students (i.e., service providers) showed higher posttest scores in positive youth development attributes, leadership attributes and life satisfaction relative to pretest scores. Besides, service providers showed positive perceptions of their learning experience, own performance, benefits to the service recipients and themselves in the SL projects. Similarly, other stakeholders also had positive evaluation of the SL projects and related benefits. Finally, trained graduate student assessors had positive assessment of the quality of program implementation. The findings underscore the utility of e-SL involving both online teaching and learning as well as online service, particularly in a Chinese context.

## Introduction

Service-Learning (SL) is a pedagogy that aims to consolidate the academic learning of the students through serving needy people and communities (Eyler & Giles, 1999). The “traditional” form of SL is to provide face-to-face service to people in need, such as teaching poor kids on personal hygiene by students of nursing programs. With the tremendous growth of online teaching programs, initiatives on developing electronic-service-learning (e-Service-Learning or e-SL) programs have also increased in recent years (Waldner et al., 2012). With the occurrence of COVID-19 in December 2019 followed by city lockdown and suspension of classes, teachers and students encountered difficulties in learning activities requiring close contact with the service recipients, such as placement and SL (Shek, 2021). Hence, e-SL has played an important role under the pandemic where community partners such as NGOs have virtually stopped “unnecessary” activities to prevent the spread of the virus.

Malvey et al. (2006) defined electronic service-learning as “an electronic form of experiential education and incorporates electronically supported service learning. It is delivered online and uses the Internet and state of the art technologies that permit students, faculty, and community partners to collaborate at a distance in an organized, focused, experiential service learning activity, which simultaneously promotes civic responsibility and meets community needs” (p. 187). According to Waldner et al. (2012), there are different modes of SL based on the dimensions of onsite versus online instruction (i.e., classroom mode versus online mode) and onsite versus online service (i.e., providing service in the field versus online). For the “traditional” mode of SL, it involves onsite instruction and onsite service. For Type I e-SL, it operates via online instruction and onsite service. For Type II e-SL, its instruction is onsite and its service is online. For Type III e-SL, it is a mixture of both online and onsite instruction and service. For Type IV e-SL, both instruction and service are online.

Faulconer (2021) highlighted several advantages of e-SL, such as lower cost and wider accessibility. Waldner et al. (2012) also pointed out that e-SL can promote student engagement in online courses. On the other hand, there are some criticisms of e-SL, such as inability of the students to experience organizational dynamics and lived experience in the community (Malvey et al., 2006). Based on a review of 24 studies on e-SL, Stefaniak (2020) highlighted several challenges in e-SL such as awareness of cultural diversity. In a recent review of Type II and Type IV e-SL studies, Faulconer (2021) pointed out that while different conceptual models were used in e-SL courses, there is a need to “better understand how to evaluate and assess eService-Learning projects” (p. 114).

## E-Service-Learning Subjects in Hong Kong under COVID-19

To provide offshore Service Learning experience for students studying at the Hong Kong Polytechnic University (“PolyU” thereafter), we have organized service-learning projects in mainland China through two SL subjects. The first subject is “Service Leadership through Serving Children and Families with Special Needs” (“Service Leadership” thereafter) that attempts to promote service leadership competencies, moral character, and caring disposition in university students. We conducted the SL projects in this subject in Chengdu and Xi’an of mainland China. In Chengdu, students typically implemented their SL projects in the form of a 5-day summer camp, with five to six university students delivering self-designed lessons to one class of around 30 children. The teaching topics included English, Science, Health Education, and Personal Development. The subject description form for this subject can be downloaded from the website (https://www.polyu.edu.hk/apss/subject/APSS2S09%20Service%20Leadership%20through%20Serving%20Children%20and%20Families%20with%20Special%20Needs.pdf).

Since the outbreak of COVID-19 in January 2020, all outbound programs were suspended. Hence, we conducted e-SL summer camps for the primary school students in 2020 and 2021. In Xi’an, we also conducted service-learning projects to serve children in need, such as migrant children and left-behind children in Xi’an. Because of the outbreak of the COVID-19 pandemic in 2020, PolyU students provided a three-day online summer camp for the students in a primary school serving urban and rural children in an underdeveloped suburban area under the administration of Xi’an. In summer 2021, our students organized a four-day online summer camp for the primary school children.

The second subject is entitled “Promotion of Children and Adolescent Development” (“Promotion” thereafter) that aims to develop university students’ general psychosocial competencies, positive values, civic responsibility, and understanding of care as well as compassion for those needy primary school students in Hangzhou. Typically, PolyU students designed and organized a five-day summer program for migrant kids at the school. For the subject description form, it can be downloaded from the website (https://www.polyu.edu.hk/apss/subject/APSS2S04%20Understanding%20Children%20in%20Poverty%20in%20Hong%20Kong.pdf). Similar to Chengdu and Xi’an, we conducted online summer camps for the primary school students in the summer of 2020 and 2021.

For students taking these two SL subjects in summer 2020 and 2021, there were several stages involved. In the first stage, students attended three online lectures to acquire the related academic knowledge (i.e., lecture classes). In the second stage, students attended four to five workshops in small groups to learn service skills, such as understanding the needs of the clients, communication skills, and online teaching skills. During this process, the students had to develop their own SL proposals and they wrote reflection assignments. In the third stage, students implemented the project in the online summer camp for around 40 h according to the SL proposals via real-time web conferencing tools. Besides, we engaged trained local graduate students in mainland China (Xi’an Jiaotong University in Xi’an, Sichuan University in Chengdu, and Zhejiang University in Hangzhou) as assessors to rate the implementation quality of the SL projects. We provided training with specific instructions for these graduate students before commencement of the service. After completion of the project, we also invited the graduate student assessors and primary school teachers to evaluate the performance of PolyU students and the quality of the service they had provided. In the final stage, university students providing the service conducted self-evaluation, made presentations on their SL projects, submitted their group presentation reports and wrote a reflective essay on their SL experiences.

It is noteworthy that these two SL subjects are award-winning subjects: “Service Leadership” was awarded the Bronze Award (Social Enterprise) in the QS Reimagine Education Award in 2016; “Promotion” was awarded the Gold Award (Sustainability) in the QS Reimagine Education Award 2017. Together with two other SL and Leadership subjects, we also obtained the UGC Teaching Award in 2018 and the Gold Award (Nurturing Student Well-Being and Purpose) in the QS Reimagine Education in 2021. As internationally renowned educators and academics are involved in the evaluation process of these teaching awards and QS awards as commonly regarded as “Oscars in Education”, it is evident that there is professional as well as academic recognition of the value and impact of these two subjects.

How might SL contribute to the holistic development of students taking the SL subjects? Theoretically, there are conceptual models proposing that SL would benefit the development of the program participants. Felten and Clayton (2011) highlighted that SL promotes civic learning, academic learning and personal growth. Regarding personal growth, Deeley (2010) pointed out that SL promotes interpersonal skills, communication, social interaction, decision making, personal confidence and social awareness, self-esteem. According to Kiely (2004), the benefits of service learning may be mediated by several personal “transformation processes” such as cognitive dissonance and reflections.

## Conceptual Framework on Evaluation

Although there are different evaluation models (Patton & Campbell-Patton, 2021), evaluators commonly agree that utilization of different evaluation models involving different stakeholders and evaluation tools is a superior form of evaluation. Through different evaluation strategies, evaluators can “triangulate” the findings based on different perspectives (Greene & McClintock, 1985). However, as commented by Kankaraš et al. (2019), “triangulation – a combined use of different assessment methods or sources to evaluate psychological constructs – is still a *rarely used assessment approach* in spite of its potential in overcoming inherent constraints of individual assessment methods (own emphasis added)” (p. 4). Hence, we employed multiple evaluation methods in this study, including objective outcome evaluation, subjective outcome evaluation based on different stakeholders and process evaluation. Actually, researchers have adopted this approach and related evaluation methods to evaluate the Project P.A.T.H.S. in Hong Kong (Shek & Sun, 2013b).

The first approach is objective outcome evaluation using objective outcome measures (Thyer & Myers, 2007). Although clinical trial is the gold standard, researchers commonly use pretest–posttest difference as an indicator of program success (Alessandri et al., 2017), including the field of education (Felix, 2014). In e-SL, studies examining change in terms of pretest and posttest scores have been conducted in the Western (Amerson, 2010; Groh et al., (2011) and Chinese (Leung et al., 2021; Lin & Shek, 2021; Shek et al., 2020) contexts.

The second approach commonly adopted is subjective outcome evaluation based on the client satisfaction approach. In fact, asking the clients and other stakeholders about their perceptions about the program quality and benefits is widely used in social work (Hsieh, 2006), education (Butt & Rehman, 2010), and allied health professions (Skar-Fröding et al., 2021). In the field of SL, educators have commonly used subjective outcome evaluation to understand the views of different stakeholders via the client satisfaction approach in Western (Chen et al., 2015; Lee et al., 2018; López-Azuaga and Suárez Riveiro, 2018) Maccio & Voorhies, 2012; Weiss et al., 2016; and Chinese studies (Leung et al., 2021; Lin & Shek, 2021).

The third evaluation approach adopted in this study is process evaluation. Compared to objective and subjective outcome evaluation, there are fewer studies on process evaluation of SL projects. Process evaluation was defined as “the use of empirical data to assess the delivery of programs …. Process evaluation verifies what the program is, and whether or not it is delivered as intended to the targeted recipients and in the intended dosage” (Scheirer, 1994, p. 40). For example, Sit et al. (2020) conducted process evaluation to understand the implementation of a large family promotion program in Hong Kong. There are several reasons why we should conduct process evaluation in SL. First, process evaluation would be helpful for researchers to understand whether all service-learning components in the SL proposals are effectively implemented (Linnan & Steckler, 2002). In addition, process evaluation could help researchers understand what happens in the program implementation process that may contribute to the outcomes (Harachi et al., 1999). Finally, process evaluation could help to prevent Type III error (i.e. a program does not have a good outcome because its implementation quality is not good). Unfortunately, Linnan and Steckler (2002) remarked that while there are many outcome studies, process evaluation studies are few. For example, Durlak (1997) reviewed 1,200 studies and found that very few studies (less than 5%) examined program implementation quality.

Based on the existing evaluation studies on e-SL, we can highlight several observations. First, few researchers used multiple evaluation mechanisms in a single study. We argue that by using different evaluation mechanisms, researchers can triangulate the evaluation findings. Second, few studies involve different stakeholders to evaluate e-SL projects. As different stakeholders (e.g., university students providing the service, service recipients and NGO staffs) may have different views, the inclusion of views held by different people would be helpful. Third, there are few studies examining online SL teaching adopting a synchronous mode and SL service conducted online. Fourth, many studies used small samples in their evaluation. Fifth, most of studies are cross-sectional studies and studies with data collected over time is almost non-existent. Sixth, there are few SL evaluation studies involving multiple sites. Finally, while the existing studies have been conducted mainly in Western contexts, published non-Western studies on e-SL are very few. With reference to these limitations in the existing SL literature, we report the evaluation findings of two e-SL subjects implemented in summer 2020 and 2021 in three sites in mainland China in this paper.

## The Present Study

Through different evaluation mechanisms, research findings suggest that these two subjects generate positive impact for the students and service recipients (Shek et al., 2020; Shek et al., 2021a, c). However, previous evaluation of SL projects in these sites has been mainly confined to face-to-face SL (Ma et al., 2019), with few studies evaluating online teaching and SL (Shek et al., 2021a, 2022). In the present study, we evaluated these two Type IV e-SL subjects (i.e., teaching and service are both conducted online) using multiple evaluation strategies based on an evaluation framework involving four mechanisms:

1. *Objective outcome evaluation involving pretest and posttest scores*: The students responded to a questionnaire covering measures of positive youth development, service leadership attributes and life satisfaction. As another colleague has used the data in other sites for another paper, we focused on the Chengdu data collected in summer 2020 and 2021 in this paper. There are four justifications for focusing on the Chengdu data. First, its sample size is not small if we compared to other similar studies on SL using pretest and posttest data. In the e-SL studies reviewed by Faulconer (2021), the sample size in the studies ranged from 14 to 46 learners. In another study on pretest and posttest differences in SL projects (Shek et al., 2020), the sample size was 138 university students. Second, in contrast to other studies that usually collected data at one time point, we collected data over two e-summer camps. Third, from a replication perspective, separation examination of the Chengdu data can help to understand the changes of PolyU students in a SL project in Southwest China as opposed to SL projects conducted in other parts of China. Finally, duplicated use of data is basically problematic.
2. *Subjective outcome evaluation of the SL programs based on the perspective of the service providers (i.e., PolyU university students)*: We examined students’ perceptions of the quality of the SL project, as well as the perceived changes in themselves and the service recipients. The importance of this from of evaluation can be seen in the remark by Kanwar and Sanjeeva (2022) that “educational institutions around the world are now requesting students’ feedback on all elements of academic life in the form of a satisfaction feedback questionnaire” (p. 1). In fact, universities are now doing student satisfaction surveys for subjects (including out of class activities) in a routine manner.
3. *Subjective outcome evaluation of SL programs based on the perspectives of other stakeholders*: We collected data from trained graduate student assessors and primary school teachers regarding their views of the SL projects after program completion. To understand the implementation quality of the SL projects, we engaged trained graduate students in mainland universities to rate the implementation quality of the teaching sessions. For primary school teachers, they provided support before and during SL project implementation (e.g., forming classes, communication with the service providers and handling student problems).
4. *Process evaluation*: To understand the implementation quality of the programs, trained graduate student assessors of the local universities rated the quality of the programs and performance of the students. Based on a structured assessment form modeled after existing measures of process evaluation on quality of program components and implementation of program activities (Centers for Disease Control & Prevention, 2018; Shek & Sun, 2013a), the assessors rated the quality of the implementation during the teaching sessions.

With reference to the gaps in the literature and the above evaluation framework, we asked the following research questions in the study:

1. Do university students (i.e., service providers) change after taking the e-SL subjects? Based on the existing theories (Felten and Clayton, 2011) and research findings on the benefits of service leadership education (Zhu & Shek, 2021b), we hypothesized that students taking the “Service Leadership” subject would change in the positive direction (Hypothesis 1). For the objective outcome evaluation measures, they are aligned with the intended learning outcomes (e.g., empathy and problem solving skills) and content of the subject (e.g., competence, character and care as service leadership qualities). We used three sets of outcome measures to assess the change involved, including indices of personal growth (indexed by the Chinese Positive Youth Development Scale), service leadership attributes and well-being (indexed by life satisfaction). These measures were also used in previous studies to measure the impact of service leadership education (Zhu & Shek, 2021b).
2. Do university students have positive perceptions of the program quality, their own performance, and benefits of SL projects (i.e., subjective outcome evaluation)? Based on previous findings (Lin & Shek, 2021), we expected that a majority of the service providers would have positive views (Hypothesis 2). It is noteworthy that numerous studies have examined descriptive profiles based on percentage data in the fields of health care (Adhikari et al., 2021; Alsaqri, 2016), education (Kanwar & Sanjeeva, 2022) and social work (Morrow-Howell et al., 1999).
3. What are the views of other stakeholders (trained graduate student assessors recruited from the local mainland universities and primary school teachers) on the service quality, performance of university students and benefits to primary school students? With reference to past studies (Shek et al., 2020), we expected that trained graduate students assessors and primary school teachers would have positive views of the e-SL programs and the service providers (Hypothesis 3). Shek and Ma (2012) pointed out that while subjective outcome evaluation studies commonly focused on the program participants, similar client satisfaction surveys were seldom conducted for other stakeholders. In this study, PolyU students were the SL subject participants whereas trained graduate student assistants and the primary school teachers were “other stakeholders”.
4. What is the quality of e-SL programs provided based on the views of the graduate student assessors (i.e., process evaluation)? Based on our previous experience and positive feedback from different stakeholders, we expected that the student assessors would have positive evaluation of the quality of program implementation (Hypothesis 4).

This study is different from Lin and Shek (2021) and Leung et al. (2021) in four ways. First, we included data collected in summer 2020 and 2021 (i.e., data cumulated over time under COVID-19) so that we can have a more complete understanding of e-SL under COVID-19. Second, besides looking at pretest–posttest change and client satisfaction findings based on the university students (i.e., service providers), we also collected subjective outcome evaluation data from trained graduate student assessors and primary school teachers (i.e., other stakeholders). Third, in the previous studies, subjective outcome evaluation focused on students’ perception of the whole subject. In the present context, we looked at students’ satisfaction with the e-SL projects. Finally, we conducted process evaluation by examining the quality of program implementation from the perspective of the graduate student assessors who observed 1,042 teaching sessions. In this paper, “PolyU students” refers to students of the Hong Kong Polytechnic University conducting the e-SL projects whereas “primary school students” were service recipients in the e-summer camps. For the “trained graduate student assessors”, we recruited them from a local university near the service site and they evaluated the implementation quality of e-SL projects after training based on assessment guidelines.

## Methods

### Participants and Procedures

For the service-learning projects in Chengdu, 82 and 85 university students participated in 2020 and 2021, respectively. They organized an online summer camp lasting for 5 days for around 500 children from a school admitting children of migrant children in summer 2020 (N = 478) and 2021 (N = 494). There were 16 classes every year, and each class was served by a group of five to six university students. For the service site in Xi’an, 41 university students served 155 pupils (94 fourth graders and 61 fifth graders) in 2020. There were seven classes of children with five to six university students serving one class. In 2021, 88 university students served 389 pupils (258 third graders and 131 fourth graders). There were 16 classes and five to six university students served one class. In the Hangzhou site, university students (N = 76) served 287 primary school students in the summer of 2020. In 2021, 292 migrant children in the serving school attended the five-day online summer camp held by 88 PolyU students. Amongst the participating students, around 27% of them were non-local students and they were motivated to take the SL subjects.

For objective outcome evaluation, students responded to the objective outcome evaluation measures at the beginning and at the end of the course. For subjective outcome evaluation on the SL projects by the service providers, students responded to the subjective outcome evaluation form after completion of the SL projects. For subjective outcome evaluation on the SL projects by other stakeholders, they completed the form after the SL projects had completed. Finally, for process evaluation, the postgraduate students received training based on an assessment guideline on how to assess the quality of the teaching sessions. For each teaching session, an assessor used one form to rate the quality of the teaching session based on their observations with reference to the items in the rating form. We have used this approach to assess the quality of program implementation in the past (Shek & Sun, 2013a).

### Instruments

#### Objective Outcome Evaluation Measures

To understand whether there were positive changes of university students after taking this subject, we employed objective outcome indicators including positive youth development attributes, service leadership qualities, and life satisfaction (see Table 1).

**Table 1:** Description of the objective outcome evaluation form for university students (i.e., service providers)

Variables	No. of Items	Sample Items
Positive youth development qualities (10 measures) (Pretest: alpha = .94; Posttest: alpha = .94)		
Social competence	3	I know how to communicate with others
Emotional competence	3	I can stand in the shoes of others
Cognitive competence	4	I know how to see things from different angles
Behavioral competence	3	I can face criticisms with an open mind
Moral competence	4	I have high moral standards about my behaviors
Self-determination	3	I am capable to make wise choices
Clear and positive identity	2	I am a person with self-confidence
Belief in the future	3	I have confidence to solve my future problems
Spirituality	4	I have found my purpose in life
Resilience	3	I believe problems in life can be solved
Service leadership qualities (4 measures) (Pretest: alpha = .97; Posttest: alpha = .98)		
Self-leadership	5	I understand the importance of self-development.
Caring disposition	8	I am sensitive to others’ needs
Character strength	15	I place my interests after the interests of others
Beliefs and values of service leadership	6	Everyone has opportunities to practice leadership every day
Life satisfaction (Pretest: alpha = .86; Posttest: alpha = .89)		
Life satisfaction	5	The conditions of my life are excellent

The outcome measures are aligned with the subject content and outcomes. First, as we covered service leadership competence in the subject, we used measures of positive youth development (PYD) attributes as outcomes (e.g., resilience, emotional competence and self-efficacy). Second, we measured other service leadership attributes, including self-leadership, caring disposition, character strengths and beliefs and values of service leadership. Finally, as we proposed that service leadership would promote well-being (Zhu & Shek, 2021b), we included life satisfaction as an outcome measure (Table 2).

**Table 2:** Changes between pre-test and post-test scores in university students (i.e., service providers; n = 140) using objective outcome evaluation

Variables	Pretest		Posttest		F	η^2^p
	Mean (SD)	α	Mean (SD)	α		
**Positive youth development qualities**	**4.61 (.59)**	**.95**	**4.94 (.54)**	**.94**	72.62^***^	**.343**
Social competence	4.82 (.67)	.91	5.21 (.61)	.91	44.58^***^	.243
Emotional competence	4.62 (.73)	.79	4.98 (.68)	.82	41.44^***^	.230
Cognitive competence	4.71 (.69)	.88	5.13 (.57)	.86	67.07^***^	.325
Behavioral competence	4.69 (.79)	.83	5.09 (.64)	.78	49.13^***^	.261
Moral competence	4.73 (.63)	.55	4.82 (.62)	.37	2.37	.017
Self-determination	4.55 (.73)	.73	4.95 (.62)	.72	51.34^***^	.270
Clear and positive identity	4.17 (.96)	.82	4.69 (.86)	.81	49.36^***^	.262
Belief in the future	4.88 (.71)	.79	5.14 (.67)	.81	24.13^***^	.148
Spirituality	4.33 (.75)	.49	4.54 (.73)	.47	14.38^***^	.095
Resilience	4.58 (.79)	.82	4.84 (.78)	.86	19.19^***^	.124
**Service leadership qualities**	**4.76 (.57)**	**.96**	**5.06 (.55)**	**.97**	61.79^***^	**.312**
Self-leadership	4.63 (.64)	.82	4.93 (.64)	.88	36.11^***^	.210
Caring disposition	4.86 (.67)	.94	5.14 (.61)	.94	33.97^***^	.200
Character strength	4.59 (.57)	.89	4.96 (.56)	.92	76.25^***^	.360
Beliefs and values of service leadership	4.93 (.64)	.91	5.23 (.66)	.95	29.35^***^	.179
**Life satisfaction**	**3.97 (.84)**	**.83**	**4.41 (.89)**	**.87**	31.85^***^	**.186**

##### Positive Youth Development (PYD) Attributes

PYD attributes were assessed using the Chinese Positive Youth Development Scale (CPYDS), which was specifically developed for Chinese youths in Hong Kong (Shek et al., 2007). There are 10 subscales of the CPYDS, including “social competence”, “emotional competence”, “cognitive competence”, “behavioral competence”, “moral competence”, “self-determination”, “clear and positive identity”, “belief in the future”, “spirituality” and “resilience”. The respondents rated each item (from 1 = *strongly disagree* to 6 = *strongly agree*), with higher scores reflecting higher levels of PYD attributes.

##### Service Leadership Qualities

Self-leadership, caring disposition, character strength, and beliefs and values of service leadership were used to measure service leadership qualities (Lin & Shek, 2021; Zhu & Shek, 2021b). Four subscales were adopted, including “self-leadership”, “caring disposition”, “character strength” and “beliefs and values of service leadership”. The respondents rated each item (from 1 = *strongly disagree* to 6 = *strongly agree*), with higher scores reflecting higher self-leadership, caring disposition, character strength, and beliefs and values of service leadership.

##### Life Satisfaction

We used the 5-item Satisfaction with Life Scale (Diener et al., 1985; Zhu & Shek, 2021b) to assess life satisfaction. The respondents responded to a 6-point Likert scale, ranging from 1 (strongly disagree) to 6 (strongly agree), with higher scores reflecting higher life satisfaction.

#### Subjective Outcome Evaluation of the SL Projects Based on University Students as Service Providers

We used a subjective outcome evaluation form (Table 3) to measure views of the university students about their service experience in the program (VP, 10 items), their own performance (VS, 8 items,), benefits of the program for service clients (BC, 9 items) and themselves (BLS, 10 items). Students rated each item on a rating scale with six response options (1 = “*strongly disagree*” for VP and VS, and “*not helpful at all*” for BC and BLS; 6 = “*strongly agree*” for VP and VS, and “*very helpful*” for BC and BLS). Higher scores represent better perceptions. This subjective outcome evaluation form was adapted from validated subjective outcome evaluation forms used in previous studies (Yu et al., 2021; Zhu & Shek, 2021a).

**Table 3:** Descriptive statistics and positive responses in subjective outcome evaluation by university students (i.e., service providers; n = 397)

		Positive Responses (5–6 Ratings)	
	Mean (SD)	*n*	%
1. Views about the service program (alpha = .95)			
1. This service program was well-designed	5.01 (.94)	305	76.8%
2. The form of the service we provided was appropriate	5.01 (.90)	314	79.1%
3. The process of delivering the service activities was pleasant	5.05 (.90)	322	81.1%
4. There was much peer interaction amongst the clients	5.10 (.87)	324	81.6%
5. The clients participated actively during our service	5.08 (.91)	320	80.6%
6. The clients were encouraged to do their best	5.22 (.77)	347	87.4%
7. We had good collaboration with different parties (school teachers, staff, etc.) during the service	5.12 (.85)	333	83.9%
8. The service experience I encountered enhanced my interest in this subject	5.05 (.93)	312	78.6%
9. The service experience increased my passion in helping people in need	5.08 (.94)	319	80.4%
10. Overall speaking, I have positive evaluation of the service program	5.14 (.89)	328	82.6%
2. Views about the service provider (alpha = .96)			
1. I prepared well for the service	5.14 (.77)	337	84.9%
2. I showed good professional attitudes in my service	5.14 (.81)	334	84.1%
3. I understood the needs and potentials of my clients	5.13 (.78)	338	85.1%
4. I was very involved in the service	5.26 (.78)	352	88.7%
5. I cared for the clients I served	5.28 (.78)	353	88.9%
6. I was ready to offer help to the clients whenever needed	5.17 (.81)	344	86.6%
7. I had much interaction with the clients	5.07 (.89)	314	79.1%
8. Overall speaking, I have positive evaluation of myself in serving the clients	5.17 (.81)	347	87.4%
3. The extent to which the service program has helped the clients (alpha = .97)			
1. It has strengthened our clients’ resilience	4.98 (.76)	299	75.3%
2. It has helped our clients face the future with a positive attitude	5.06 (.77)	305	76.8%
3. It has improved our clients’ self-confidence	5.03 (.78)	309	77.8%
4. It has broadened our clients’ horizon	5.20 (.74)	340	85.6%
5. It has reinforced our clients’ interest in learning	5.09 (.76)	327	82.4%
6. It has strengthened our clients’ ability to care for other people	4.99 (.81)	297	74.8%
7. It has helped our clients build up good relationships with healthy adults	5.10 (.75)	328	82.6%
8. It has promoted our clients’ aspirations in life	5.04 (.77)	309	77.8%
9. It has enriched the overall development of our clients	5.11 (.73)	328	82.6%
4. The extent to which the service learning program has helped the service provider (alpha = .96)			
1. It has enabled me to understand the needs and potentials of the service recipients	5.09 (.75)	319	80.4%
2. It has helped me integrate the academic knowledge into real life situation through service delivery	5.04 (.81)	309	77.8%
3. It has enhanced my competences in problem-solving and decision-making	5.15 (.71)	329	82.9%
4. It has helped me appreciate and respect people from diverse background	5.17 (.74)	333	83.9%
5. It has helped me develop the sense of care and compassion towards other people	5.19 (.77)	331	83.4%
6. It has improved my interpersonal skills	5.15 (.79)	326	82.1%
7. It has boosted my self-confidence	5.01 (.82)	304	76.6%
8. It has enabled me to apply the knowledge and skills I acquired in university to solve complex issues in the service	5.01 (.85)	308	77.6%
9. It has inspired me to reflect on my roles and responsibilities as both a professional and a responsible citizen	5.06 (.79)	320	80.6%
10. On the whole, I am satisfied with this service program	5.09 (.84)	326	82.1%

#### Subjective Outcome Evaluation of SL Projects Based on the Perspectives of Other Stakeholders

Both trained graduate student assessors and primary school teachers evaluated the quality of the SL projects after project completion. The assessment tool contains a structured questionnaire and two open-ended questions (Table 4). The service partners reported their perceptions of three aspects of service project, including qualities of service project (from 1 = *strongly disagree* to 6 = *strongly agree*), performance of service providers (from 1 = *strongly disagree* to 6 = *strongly agree*) and perceived benefits for service recipients (from 1 = *not helpful at all* to 6 = *very helpful*) on a 6-point Likert scale. We computed mean score for each dimension, with a higher score reflecting better evaluation of the service project.

**Table 4:** Descriptive statistics and positive responses based on post-service evaluation by trained graduate students assessors and primary school teachers (n = 113)

		Positive Responses (5–6 Ratings)	
	Mean (SD)	*n*	%
1. Evaluation about the program (alpha = .91)			
1. This service program was well-designed	5.68 (.63)	112	99.1%
2. The form of the service we provided was appropriate	5.54 (.72)	107	94.7%
3. The process of delivering the service activities was pleasant	5.61 (.72)	106	93.8%
4. The service met the needs of the service recipients	5.63 (.54)	110	97.3%
5. The service promoted the positive atmosphere of the community (e.g., school)	5.64 (.54)	110	97.3%
6. My observation and understanding of this service promoted my interest in related programs	5.68 (.50)	111	98.2%
7. Overall speaking, the service was beneficial to the service recipients	5.72 (.47)	112	99.1%
8. Overall speaking, I have positive evaluation of the service program	5.78 (.44)	112	99.1%
2. Evaluation about the PolyU students’ Performance (alpha = .92)			
1. PolyU students prepared well for the service	5.79 (.45)	111	98.2%
2. PolyU students showed good professional attitudes in their service	5.76 (.45)	112	99.1%
3. PolyU students understood the needs and potentials of their clients	5.63 (.55)	109	96.5%
4. PolyU students were very involved in the service	5.81 (.39)	113	100%
5. PolyU students cared for the clients they served	5.79 (.43)	112	99.1%
6. PolyU students were ready to offer help to the clients whenever needed	5.81 (.39)	113	100%
7. PolyU students had much interaction with the clients	5.69 (.55)	110	97.3%
8. Overall speaking, I have positive evaluation of PolyU students in serving the clients	5.79 (.40)	113	100%
3. Benefits towards the clients (i.e., our primary school children) (alpha = .96)			
1. It has strengthened the clients’ resilience	5.47 (.68)	101	89.4%
2. It has helped the clients face the future with a positive attitude	5.60 (.59)	107	94.7%
3. It has improved the clients’ self-confidence	5.69 (.57)	107	94.7%
4. It has broadened the clients’ horizon	5.74 (.49)	110	97.3%
5. It has reinforced the clients’ interest in learning	5.65 (.56)	108	95.6%
6. It has strengthened the clients’ ability to care for other people	5.51 (.68)	101	89.4%
7. It has helped the clients build up good relationships with healthy adults	5.54 (.68)	101	89.4%
8. It has promoted the clients’ aspirations in life	5.60 (.64)	102	91.9%
9. It has enriched the overall development of the clients	5.64 (.58)	107	94.7%
10. On the whole, I am satisfied with this service program	5.77 (.46)	111	98.2%

#### Process Evaluation

To understand the quality of the e-SL implementation process, a trained graduate student assessor rated the quality of the teaching sessions on a five-point scale with response options ranging from “very good”, “good”, “fair” and “poor” to “very poor”. The rating items include different aspects of service quality, such as care for the service recipients, cultural sensitivity, and overall performance (see Table 5).

**Table 5:** Descriptive statistics and positive responses based on observations by graduate student assessors recruited from mainland universities (n = 1,042 teaching sessions)

			Positive Responses (4–5 Ratings)	
	α	Mean (SD)	*n*	%
**Field Observation**	**.84**			
1. Service Attitude		4.89 (.33)	1035	99.3%
2. Sense of Responsibility		4.89 (.34)	1029	98.8%
3. Awareness of Needs		4.52 (.69)	958	91.9%
4. Sense of Care		4.72 (.55)	1005	96.4%
5. Cultural Sensitivity		4.28 (1.17)	917	88.0%
6. Teamwork		4.62 (.82)	989	94.9%
7. Problem Solving Skills		4.46 (.81)	948	91.0%
8. Communication Skills with service targets		4.58 (.64)	962	92.3%
9. Teaching Skills		4.51 (.65)	971	93.2%
10. Application of discipline-related Knowledge		4.65 (.66)	983	94.3%
11. Reflective Attitude		4.63 (.87)	978	93.9%
12. Punctuality		4.79 (.52)	997	95,7%
13. Overall Performance		4.69 (.50)	1014	97.3%

## Results

Based on the pretest and posttest data collected in Chengdu in 2020 and 2021, we conducted MANOVAs to examine changes in the university students (Table 2). For measures of positive youth development qualities, as omnibus F value was significant, we performed univariate ANOVA analyses. Results showed that the students showed significantly higher scores on all measures (except moral competence) at posttest. We also found similar positive changes on service leadership qualities and life satisfaction after joining the SL project (p < 0.01). Overall speaking, these findings provided support for Hypothesis 1.

For subjective outcome evaluation based on the university students (i.e., service providers), more than three-quarters of the respondents showed positive perceptions of the service program, service provider, benefits to the clients and benefits to oneself (Table 3). For example, around 90% of the respondents responded that they cared about the clients they served and around 83% of the university students agreed that the e-summer camp enriched the overall development of the primary school students. Besides, 83% of the students agreed that the SL program promoted their “care and compassion toward other people”. The findings generally supported Hypothesis 2.

For the subjective outcome evaluation findings based on trained graduate student assessors and primary school teachers, there is similar support for Hypothesis 3 (Table 4). In the areas of program quality and performance of the service providers, nearly all respondents gave positive responses. For perceived benefits to the primary school students, around 95% of the respondents agreed that the SL program promoted the overall development of the clients and around 95% of them agreed that the program had improved the self-confidence of the service recipients.

Regarding the implementation quality of SL projects (i.e., process evaluation), results showed that the perceived service quality was high. Nearly all respondents agreed that service attitude and responsibility of the students were good or very good; 94% of the responses to the items on knowledge application, reflection and teamwork were good. As a whole, 97% of the respondents regarded the overall performance of the service to be good and very good. These findings gave support to Hypothesis 4.

## Discussion

Although there is a growing trend to conduct e-SL projects, systematic evaluation studies are rare. As pointed out by Figuccio (2020), “E-service-learning is a relatively new pedagogical practice … Unlike service-learning, however, e-service-learning has not been extensively studied and evaluated” (p. 2). Similarly, Faulconer (2021) remarked that their review “presents a clear call to research – one that aims at resolving unknowns within eService-Learning” (p. 100).

With reference to the limitations of the existing evaluation studies on e-SL outlined earlier in the paper, this study has several advances. First, we used multiple evaluation strategies to assess the effectiveness of e-SL programs. These included objective outcome evaluation, subjective outcome evaluation of the SL projects based on university students’ perspective (i.e., service providers) and other stakeholders’ perspectives (trained student assessors and primary school teachers), and process evaluation on the quality of program implementation. These different sources of data can help to triangulate the evaluation findings across different types of data. Second, we engaged different stakeholders, including service providers (i.e., university students), trained graduate student assessors and primary school teachers. Again, this helps to triangulate the evaluation findings across different stakeholders. Third, as there are very few studies adopting Type IV e-SL mode, we focused on Type IV e-SL programs. Fourth, in contrast to the existing studies with small samples, we collected a large number of subjective outcome and process evaluation forms. Fifth, unlike most existing studies involving “one-shot” data, we collected data over the summer of 2020 and 2021. This can help to consolidate the evaluation findings over time. Sixth, we collected data over multiple sites instead of a single site. Finally, we collected data from a non-Western context. As far as the generalizability of the findings are concerned, the present study showed that the observations were generalized over time, site and culture based on data collected via different methods and stakeholders.

For the change in the university students (i.e., service providers), the findings support Hypothesis 1. These findings triangulated the previous studies based on face-to-face SL (e.g., Shek et al., 2020). The positive changes occur in three areas in this study. First, the students showed positive change in PYD attributes (except moral competence). As the subject covered leadership competence (e.g., adversity and problem solving skills) and the students actually practiced the related skills in the process (such as improvised under changing conditions of the pandemic), this can explain why the change is in the positive direction. Second, the students showed positive change in service leadership beliefs and values, character and care. Again, as we covered the attributes of service leadership in the subject and the students had opportunities to practice and reflect on their leadership qualities (e.g., care about the service recipients and doing the SL project in a responsible manner), this can explain for the positive change. Finally, life satisfaction of the students also changed in the positive direction. In the Service Leadership Theory and previous studies using cross-lagged analyses (Zhu & Shek, 2021b), we proposed that service leadership attributes positively promoted the well-being of the students. Nevertheless, despite these positive findings, as there was no control group, other alternative explanations such as maturation may explain the positive change. Besides, as we did not assess the transformational processes in the process of “change” in the students (Kiely, 2004), future studies should explore this area. One possibility is to analyze the reflections of the students but this is beyond the scope of this study.

For subjective outcome evaluation of the SL projects based on the service providers and other stakeholders, the findings are also positive. Using percentage data, the findings suggest that the SL programs were beneficial to the service recipients and the university students themselves. Of course, subjective outcome evaluation may be criticized as “too subjective”. However, we have three counter-arguments to this criticism. First, looking at client satisfaction profile is a very common approach in human services evaluation, including social work, education and allied health disciplines. Second, researchers and practitioners have regarded client satisfaction survey as an important approach of evaluation. In the area of health care, Comans et al. (2011) remarked that “the most reported outcome related to patients was satisfaction surveys” (p. 19). In the area of education, Santini et al. (2017) pointed out that student satisfaction had been widely examined in the past three decades. Third, studies showed that objective outcome evaluation and subjective outcome evaluation were actually correlated significantly (Shek, 2010, 2014).

For process evaluation, the findings showed that the implementation quality of the e-SL programs were generally positive, particularly the sense of care, responsibility, teaching skills, problem solving skills, cultural competence, application of discipline-specific knowledge and reflection amongst the students. There are three unique aspects of the findings. First, as there is no published study on process evaluation on e-SL, this is a pioneer study. Second, we prepared an assessment guideline for the graduate students. Third, the trained graduate student assistants rated a large number of teaching sessions. According to Limbani et al. (2019), “process evaluation is increasingly recognized as an important component of effective implementation research and yet, there has been surprisingly little work to understand what constitutes best practice” (p. 1). Hence, this study has contributed to our understanding of the implementation quality of the SL projects. In future, researchers can consider using more items on the quality of the implementation process (Shek & Sun, 2013a, b). Besides, we should involve more raters to ensure inter-rater reliability. In the present context, we were not able to involve more raters because of the pandemic.

Compared with the findings based on Lin and Shek (2021) and Leung et al. (2021), the present study generates several areas of “new knowledge”. First, we utilized data collected in summer 2020 and 2021 (i.e., the data under examination are not just based on a “one-shot” occasion). As we collected data over two years, this can give us a more stable picture about the impact of e-SL programs. Second, from a replication perspective, the present findings replicated the findings reported in these two studies based on aggregation of data over time. Third, while the previous two studies examined subjective outcome evaluation of the *subjects*, we examined students’ views on the *e-SL projects*. Fourth, besides looking at pretest–posttest change and client satisfaction findings based on the university students (i.e., service providers), we also collected subjective outcome evaluation data on SL projects from trained graduate student assessors and primary school teachers (i.e., other stakeholders). This is very important because triangulation is an important principle in evaluation. Finally, we conducted process evaluation by examining the quality of program implementation from the perspective of the graduate student assessors who observed 1,042 teaching sessions.

There are several theoretical implications of the present findings. First, this study illustrates the positive impact of e-SL that is relatively unexplored in the existing scientific literature. In future, we should also examine the impact of e-SL highlight in the SL literature, such as self-understanding, learning attitudes, academic achievement, social competence and civic engagement (Celio et al., 2011). Besides, other models on human behavior may provide insights on the possible developmental outcomes of SL. For example, with reference to the bio-psychosocial-spiritual model (Sulmasy, 2002), spiritual domain is an important but neglected study. In this study, we found that spirituality in students increased after joining the SL subject. In future, it would be theoretically important to see whether e-SL would promote life meaning (such as engaging in prosocial behavior) in the students taking the SL subject. Besides, it would be theoretically interesting to use different quality of life models (Felce & Perry, 1995; Wallander et al., 2001) to ask how e-SL may shape the quality of life in various domains in different stakeholders.

Second, as there are few studies on the development of late adolescents and emergent adults in mainland China (Shek et al., 2021b), the present findings suggest that SL as a pedagogy works in the Chinese context. Of course, there is a need to replicate the present findings in different Chinese societies in future. Third, this study highlights the value of employing an evaluation framework involving multiple evaluation strategies. In the discussion of evaluation research, Patton and Campbell-Patton (2021) argued that there are different approaches in looking at the impact of a program, particularly the quantitative and qualitative approaches. Obviously, it would be helpful to examine the impact of e-SL programs such as using a case study approach.

There are several practical implications of the findings. First, researchers and workers can make use of e-SL to serve communities that are not accessible, such as those in the mountain or desert areas. Second, the study highlights the feasibility and value of conducting process evaluation in SL. In the scientific literature on SL, although quantitative studies (Celio et al., 2011; Conway et al., 2009; Eyler et al., 2001; Salam et al., 2019; Yorio & Ye, 2012) and qualitative studies (e.g., Meili et al., 2011; Steinberg et al., 2010) highlighted the positive outcomes of SL, there are very few studies on the process of implementing e-SL. Hence, researchers should step up process evaluation effort. Scott et al. (2019) criticized that “most process evaluation data collection occurred post-intervention undermining the ability to evaluate the *process* of implementation” (p. 1). Finally, the study highlights the importance of engaging different stakeholders in understanding the value of e-SL.

While the present evaluation findings are pioneer and positive regarding Type IV e-SL programs, we have to acknowledge several limitations of this study. First, although university students showed positive changes over time, no control group was used. The non-inclusion of a control group obviously raises the question of alternative explanations such as natural maturation. Second, although the number of rating forms collected under subjective outcome evaluation by university students and process evaluation is large, the number of evaluation forms based on the other stakeholders is not high. This may be due to the fact that it was difficult to engage the trained graduate student assessors and primary school teachers after completion of the SL programs. Third, we should understand the limitations of subjective outcome evaluation approach to evaluation, despite its widespread use in different disciplines. Fourth, more items can be added to the process evaluation rating form, such as the classroom interaction and management of the service providers. Effort to involve more raters in the process would also be helpful. Fifth, while the quantitative findings based on the four evaluation mechanisms are positive, it would be helpful to collect qualitative data to understand the lived experiences and underlying mechanisms involved (Patton & Campbell-Patton, 2021). In particular, it is important to understand the “transformational processes” taking place during service learning. Despite these limitations, the present study provides pioneer evidence for the usefulness of Type IV e-SL subjects with both online synchronous teaching and online SL service.
